# Pathological protection and molecular regulation of cystatin B in Nile tilapia (*Oreochromis niloticus*) bacterial disease

**DOI:** 10.1080/21505594.2025.2563010

**Published:** 2025-09-29

**Authors:** Yongxiong Huang, Xuyan Tan, Yuwen Li, Zhiqiang Zhang, Yu Huang, Jichang Jian

**Affiliations:** a Guangdong Provincial Key Laboratory of Aquatic Animal Disease Control and Healthy Culture & Key Laboratory of Control for Disease of Aquatic Animals of Guangdong Higher Education Institutes, Fisheries College of Guangdong Ocean University, Zhanjiang, China; b Guangdong Provincial Engineering Research Center for Aquatic Animal Health Assessment, Shenzhen Institute of Guangdong Ocean University, Shenzhen, China

**Keywords:** Cystatin B, *Oreochromis niloticus*, inflammation, pyroptosis, *Streptococcus agalactiae*, Aeromonas hydrophila

## Abstract

Cystatin B (CSTB) is an endogenous cysteine protease inhibitor that plays a critical role in the modulation of numerous biological processes, involving immune responses, apoptosis, and inflammation in mammals. However, its immunological functions in teleost, particularly Nile tilapia (*Oreochromis niloticus*) remain unclear. In the present study, the *CSTB* was cloned and characterized from Nile tilapia (*On-CSTB*) and its role in bacterial infection was revealed. The On-CSTB open reading frame is 300 bp encoding 99 amino acids that contains a conserved cystatin domain. Multiple sequence alignment analysis revealed that On-CSTB share over 75% identity with fish lineages, and 45% identity with mammals. qPCR analysis showed that *On-CSTB* is widely expressed across various tissues and highly expressed in blood cells and intestines and can be significantly inducted by *Streptococcus agalactiae* (*S. agalactiae*) and *Aeromonas hydrophila* (*A. hydrophila*). In vivo experiments demonstrated that On-CSTB protein could suppress inflammation while participating in the MyD88/NF-κB signaling pathway and inflammasome activation, affecting apoptosis and pyroptosis processes. Moreover, On-CSTB contributed to enhanced tissue integrity and alleviated pathological damage. These findings collectively highlight On-CSTB as a crucial immunomodulator that contributes to host defense and tissue protection in fish. The present study offers novel perspectives on the immunomodulatory role of CSTB in tilapia, providing a basis for disease resistance strategies in aquaculture.

## Introduction

Cystatins are a super family of cysteine protease inhibitors that ubiquitously exist in plants to animals, functioning primarily by inhibiting the proteolytic activity of cysteine proteases and participating in diverse physiological processes, such as immune regulation and apoptosis [1,2]. Cystatins are categorized into four distinct families according to their characteristic amino acid sequences. Among them, Family 1 (stefins), including cystatin B, are intracellular inhibitors lacking disulfide bonds and glycosylation [3–6].

Cystatin B (CSTB), initially identified as a cathepsin B inhibitor in humans, is predominantly localized in the cytoplasm and nucleus, and can also be detected extracellularly [2,7]. Recent findings suggest that cystatin B fulfills a pivotal function in immune responses and neurodegenerative diseases, while also exhibiting antibacterial, antiviral, antifungal, and anti-apoptotic properties [8–11]. Cystatin B has been shown to play conserved immunomodulatory roles across species. In mammals, CSTB-deficient mice display heightened susceptibility to lipopolysaccharides (LPS)-induced sepsis and enhanced noncanonical inflammasome activation in macrophages [12,13]. In invertebrates such as hirudo (*Theromyzon tessulatum*), CSTB expression is upregulated upon bacterial challenge [14]. Similarly, in teleost, CSTB has been reported to promote macrophage-mediated bacterial clearance in turbot (*Scophthalmus maximus*) [8], and to exhibit bacteriostatic activity against gram-negative bacteria in striped beakfish (*Oplegnathus fasciatus*) and red piranha (*Pygocentrus nattereri*) [15,16]. Nonetheless, the functions of cystatin B in fish are still limited in immunomodulation mechanisms, especially defense in invasion of pathogens. Although cystatin B has been reported to regulate immune and inflammatory responses in mammals, its immunomodulatory roles in teleost remain largely unknown. Particularly, the potential involvement of CSTB in the MyD88/NF-κB signaling cascade and inflammasome activation has not been elucidated in Nile tilapia (*Oreochromis niloticus*). Given the importance of these pathways in regulating apoptosis and pyroptosis, understanding CSTB function is essential for advancing our knowledge of fish immunology. Therefore, this study aimed to characterize the function of Nile tilapia CSTB in inflammatory regulation and determine its involvement in key immune signaling pathways during bacterial infection.

The Nile tilapia is a farmed fish recommended by the food and agriculture organization [17] for its fast growth, strong adaptability to the environment, high yield, and good meat quality, and is widely cultivated worldwide, especially in China [18,19]. However, some nonstandard farming, especially high-density farming, and the abuse of antibiotics lead to frequent outbreaks of bacterial diseases, causing setbacks to the farming industry. *Streptococcus agalactiae* and *Aeromonas hydrophila* are two major pathogenic bacteria in Nile tilapia causing huge deaths of fish and without effective treatment. Therefore, further studies are needed to fully elucidate the immune functions of cystatin B. In this study, *cystatin B* was cloned from *Oreochromis niloticus* (On-CSTB) and the role of cystatin B was characterized during *S. agalactiae* and *A. hydrophila* infection. The findings will provide valuable insights into the immunological function of cystatin B in teleost.

## Materials and methods

### Fish preparation

The fish (50 ± 5 g) were purchased from a tilapia farm in Zhanjiang, Guangdong province. Then the fish were reared in aquariums at a condition of 60 fish per 1,000 liters for over a month. And the fish were provided with commercial feed with an amount of 3% weight of fish each day. Water quality parameters were maintained as follows: temperature, 28 ± 0.5°C; pH, 7.4–7.6; and dissolved oxygen levels, 5.0–6.5 mg/L, as previously described [20].

### RNA extraction and cDNA synthesis

The fish tissues including blood cells, liver, brain, spleen, head kidney, muscle intestine, skin, and gill were collected after fish were anesthetized with clove oil (Sigma, Darmstadt, Germany). RNAiso Plus (TaKaRa, Dalian, China) was used to extract total RNA immediately. The total RNA was reversed in complementary DNA (cDNA) using PrimeScriptTM RT reagent kit with gDNA Eraser (TaKaRa, Dalian, China). In brief, genomic DNA was first eliminated from total RNA via gDNA Eraser treatment (42°C, 2 min), followed by reverse transcription of the RNA into cDNA using the provided reagent mix (37°C, 15 min, followed by 85°C, 5 sec).

### Cloning and characterization of cystatin B

The gene *cystatin B*(*CSTB*) (genBank accession number: XM_003443609.5) open reading frame (ORF) was predicted using NCBI ORF Finder, and gene-specific primers (Table 1) were designed using Primer 5.0 software. Polymerase chain reaction (PCR) amplification was performed under the following conditions: initial denaturation at 95°C for 5 min; 29 cycles of 95°C for 15 s, 58°C for 15 s, and 72°C for 30 s; followed by a final extension at 72°C for 5 min and the cloned products were purified, linked to the T vector and transformed into the competent cell (TransGen, Guangzhou, China), and the monoclonal colonies were identified and then sequenced were identified and subsequently sequence. Homologous sequence alignment of cystatin B was performed using DNAMAN software (version 10.0, USA). The phylogenetic tree was facilitated by the utilization of MEGA 7.0.26 software. Signal peptide was predicted using SignalP (https://services.healthtech.dtu.dk/services/SignalP-5.0/). The molecular weight of cystatin B was predicted by ProtParam (https://web.expasy.org/protparam/).

**Table 1.** Primer sequences and target genes.

Genes	Primer name		Sequence (5′–3′)
CSTB	CSTB-F1	ATGCCGATGTGCGGAGGGC	
	CSTB-R1	TTAGAAGTACTCAATACTGTCATCA	
	On-CSTB-F	CGCGGATCCATGCCGATGTGCGGAGGGCT	
	On-CSTB-R	CCGGAATTCGAAGTACTCAATACTGTCATCATGA	
qRT-PCR			
CSTB	qCSTB-F1	ATGTGCGGAGGGCTGACTG	
	qCSTB-R1	CGGTTTTCTGCTCTGCGTGA	
IL-1β	IL-1β-F	CAGTGAAGACCGCAAAGTGC	
	IL-1β-R	CGGTCTCCTGACACACTTCC	
TNFα	TNFα-F	ATGTGAGAGCAGCCATTCAT	
	TNFα-R	ACAAAGTAGAGGCCATCTCG	
IL-10	IL-10-F	GCTTCCCCGTCAGGCTCAA	
	IL-10-R	CTGTCGGCAGAACCGTGTC	
TGF-β1	TGFβ1-F	TGGGACTATGAGCAGGAGGG	
	TGFβ1-R	AACAGCAGTTGTGTGATTGGGT	
P65	P65-F	AAGAACGCACCCTACAAGCC	
	P65-R	TCCAGAGGATAGAGCTCCCC	
P38	P38-F	CTGCTCAAACACATGAAGCA	
	P38-R	AGCCTCTGAGGATCTGGTAT	
MyD88	MyD88-F	AAGATTCCTCTCGTTGCGCT	
	MyD88-R	CAACTTCCCCACTGACGTGT	
Caspase-3	Caspase3-F	GCAAAGCCAGGCAATGGAAA	
	Caspase3-R	TCCACGTCAGTACCGTTTCG	
Caspase-9	Caspase9-F	CTGTATCATGGGAGGAGGAGCAA	
	Caspase9-R	TTGACCTGAGGGCTCGCTTC	
Bcl2	Bcl2-F	CTGTACCAGCCGGACTTCAC	
	Bcl2-R	CCACAAATGCATCCCATCCC	
Caspase-1	Caspase-1-F	CCTGCACTCCAGATACTGTGT	
	Caspase-1-R	TTTGTTGGCATCTGTCTCCTGT	
ASC	ASC-F	CGCGTAAAACGCAACAAGGT	
Gsdme	ASC-R Gsdme-F	TCTCGAGAACCACGGTCAGA AGCAAAGACGGGTATTTGGGAT	
	Gsdme-R	CCCAAGCTTGCGTTCTCCTT	
NOD1	NOD1-F	GTGGCTTACCCCACTACGAC	
	NOD1-R	GAGCCCCAGGAGTTTCTGAC	
NOD2	NOD2-F	CCCAGCGCCATCAAAATCTG	
	NOD2-R	AAAGGCATCTTGCCACCTGT	
NLRC-3	NLRC-3-F	TCAGCTGCCCCAGACTTAAC	
	NLRC-3-R	AGACCCTAGGGGGCATACTG	
NLRP-3	NLRP-3-F	GGAGAAGAGGAGCAAGCCAG	
	NLRP-3-R	TGGATTCCAGTGTGGTTCCG	
NLRP-14	NLRP-14-F	GGGACTGAACGGTACCACTG	
	NLRP-14-R	AGGCCAAACATGAAACGAAGT	
β-actin	β-actin-F	AGATGAAATCGCCGCACTGG	
	β-actin-R	TCTGACCCATACCCACCATCA	
GAPDH	GAPDH-F	CCGTTACCGTGGTGAAGTGT	
	GAPDH-R	AACATTGGAGCATCGGGTGA	

### Acute bacterial infection

The bacteria of *S. agalactiae* (ZQ0910) [21] and *A. hydrophila* [22] both isolated from tilapia, were grown separately in brain-heart infusion (BHI) and luria-bertani (LB) liquid media at 28°C for 12 h. And the bacteria were collected, washed three times, and resuspended in phosphate-buffered saline (PBS) to achieve final concentrations of *S. agalactiae* (5 × 10^7^ colony-forming unit (CFU)/mL) and *A. hydrophila* (5 × 10^7^ CFU/mL). 120 fish were divided into two groups, with each group receiving an intraperitoneal injection of 100 μL/fish of either *S. agalactiae* or *A. hydrophila*. At five different time points (0, 6, 12, 24, and 48 hours), three fish from each group were sacrificed, and the brain, spleen, liver, head kidney, and intestine were collected. The extraction of RNA and the synthesis of cDNA were carried out immediately afterward.

### Quantitative real-time PCR (qRT–PCR)

Tissue distribution of *cystatin B* in normal fish and the expression profile following bacterial infection were assessed using QuantStudio 6 Flex Real-Time PCR Systems (Thermo Fisher Scientific, Waltham, USA). *Glyceraldehyde-3-phosphate dehydrogenase* (*GAPDH*) and *β-actin* served as internal reference genes, and the genes relative expression were calculated using the methods described by Vandesompele [23] and Hellemans [24].

### Preparation of recombinant protein

The special clone primers linked with restriction sites were used to amplify *cystatin B*. and clone fragments were sequencing and linked with pGEX-4T-1 vector (BT Lab, Wuhan, China) and transformed into competent cells BL21. The positive clone was cultured in LB medium added with ampicillin (100 ug/mL) at 37°C and the 0.5 mmol/L of isopropyl β-D-thiogalactoside was added when the optical density (OD) reached an absorbance 0.4–0.6 at OD 600 nm. The induction was performed at 16°C for 12 h to enhance soluble protein folding. The mixture of bacteria was collected and recombinant cystatin B was purified by GST Purification Kit (Takara, Dalian, China). And the cystatin B was confirmed using sodium dodecyl sulfate polyacrylamide gel electrophoresis (SDS PAGE) and western blot. For glutathione s-transferase (GST)-tagged protein, the entire pGEX-4T-1 plasmid was also transfected into competent cells BL21 and tag protein was purified as mentioned above.

### Western blotting

The purified recombinant GST and CSTB proteins were isolated using SDS – PAGE and then transferred to a polyvinylidene fluoride (PVDF) membrane (Thermo Fisher Scientific, Waltham, Massachusetts, USA) by Trans-Blot Turbo system (Bio-Rad, California, USA). After transfer, membrane was then incubated in Western Blot Blocking Reagents (Bio-Rad, California, USA) for 30 minutes. GST tag monoclonal antibody (Thermo Fisher Scientific, Shanghai, China) was applied for 1 hour. After incubation, the membrane was washed three times with tris-buffered saline with tween 20 (TBST) and incubated for 40 minutes with goat anti-mouse immunoglobulin G (IgG) (SAB, Nanjing, China). Following additional washing with TBST, protein detection was performed by the Clarity Western ECL Substrate Kit (Bio-Rad).

### Cystatin B function of tilapia against bacterial infection assay

Fish were randomly divided into seven treatment groups (*n* = 50 per group), including PBS group, *S. agalactiae* + CSTB group, *S. agalactiae* + GST group, *S. agalactiae* + PBS group, *A. hydrophila*+ CSTB group, *A. hydrophila* + GST group, and *A. hydrophila* + PBS group. *S. agalactiae* (5 × 10^7^ CFU/mL) and *A. hydrophila* (5 × 10^7^ CFU/mL) were prepared as above. Each fish in the *S. agalactiae* + PBS group and *A. hydrophila* + PBS group was injected with 100 µL of *S. agalactiae* or *A. hydrophila* suspension, respectively. For the recombinant protein groups, each fish received a co-injection of 100 µL bacterial suspension along with 80 µg of either GST or CSTB recombinant protein [25]. The collected tissues included brain, liver, intestine, head kidney, and spleen were sampled at five timings (0, 6, 12, 24, and 48 hour(h)) for cDNA detection from each group. At each time point, three fish per group were used as biological replicates. Additionally, three fish from the PBS group at 0 h were sampled as the mock control (Mock). Daily mortality was recorded, and survival rates (SR) were calculated based on previously established methods [26].

$$\mathrm{SR}=\;\;\;\;\;\;\;\;\left(1-\frac{\mathrm{Dead}\;\mathrm{fish}}{\mathrm{Survivalfis}h_{\left(\mathrm{previous}\;\mathrm{day}\right)}\mathrm{sampled}\;\mathrm{fish}}\right)\times SR_{\left(\mathrm{previous}\;\mathrm{day}\right)}\times 100\%$$

### Histology analyses

The tissues (spleen, liver, and head kidney) were sampled from normal fish and challenged fish, and fixed in Dietrich’s fixative for over 24 h. After fixation, the tissues were dehydrated by gradient ethanol (75%–100%), followed by clearing in xylene and then immersed in liquid paraffin and embedded in paraffin. The tissue sections (6 µm thickness) were prepared using an automated rotary microtome (Leica RM2235, Germany). Subsequently, the sections were first dewaxed using xylene and then rehydrated through a gradient series of ethanol solutions (100%–75%), and stained using Hematoxylin-Eosin (H&E) Stain Kit (Solarbio, Beijing, China). Stained sections were imaged using a Nikon DS-Ri2 microscope (Nikon, Tokyo, Japan).

### Statistics and illustrations

Adobe Photoshop CC (San Jose, CA, USA) was utilized for visual creation and figure panel arrangement. Data are presented as means ± standard deviations (SD). Statistical analyses were performed using Prism software (Version 8.0), with one-way ANOVA followed by Tukey’s HSD test and Student’s *t*-test applied to assess significant differences. A significance threshold of *p* < 0.05 was considered statistically significant.

## Results

### Characteristics of On-CSTB

**Figure 1.**
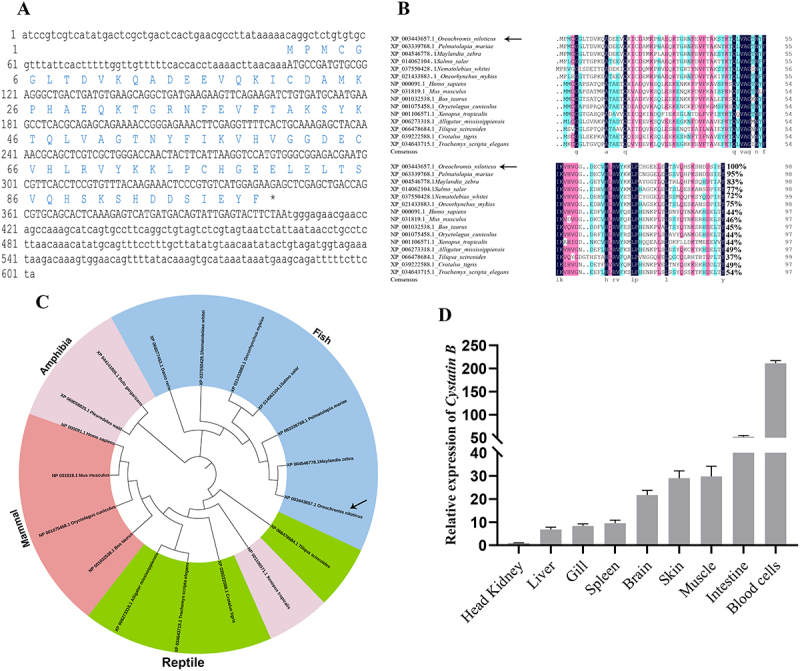
(A) Nucleotide and predicted amino acid of On-CSTB, with the ORF shown in uppercase letters. (B) Cross-species multiple sequence alignment of the CSTB. (C) Phylogenetic analysis of the CSTB family across species. (D) *On-CSTB* transcript levels in various tissues via qRT-PCR. Data are presented as mean ± SD (*n* = 3).

The *On-CSTB* open reading frame (ORF) is 300 bp and the predictive protein codes 99 amino acids (Figure 1A) without signal peptide. The molecular weight of On-CSTB is 11.2 kDa and a theoretical isoelectric point of 6.03. Multiple sequence alignments revealed On-CSTB contain a conservative CSTB domain. Basic local alignment search tool (BLAST) analysis revealed that On-CSTB shares high sequence homology with CSTB from other species, exhibiting over 75% identity with fish lineages, approximately 45% identity with mammals, and 37% to 54% identity with amphibians and reptiles (Figure 1B). Phylogenetic tree revealed that On-CSTB first clustered with the fish species, followed by clustering with those from amphibians, reptiles, and mammals (Figure 1C).

### Expression characteristics of On-CSTB among various tissues

On-CSTB expression in various tissues was assessed via qPCR. The expression of On-CSTB was highest in blood cells, followed by the intestine and the lowest tissues was head kidney. Meanwhile, the relative expression of *On-CSTB* in blood cells and intestine was up to 200-and 50-fold, respectively, compared with head kidney (Figure 1D).

### On-CSTB expression pattern post bacterial challenge

**Figure 2.**
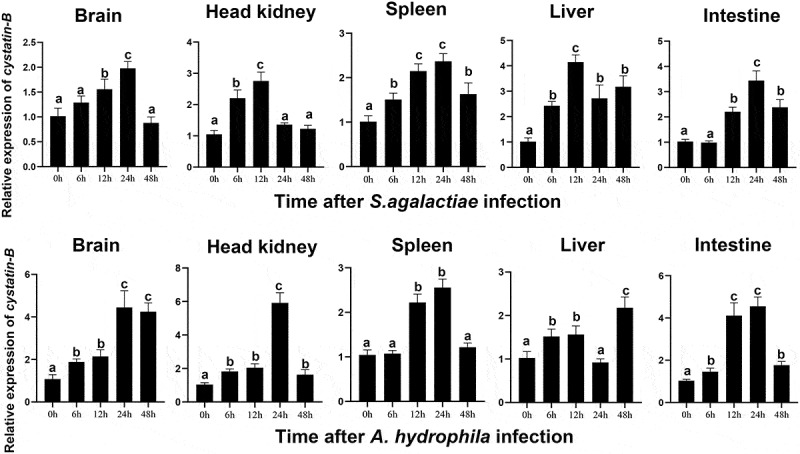
*On-CSTB* expression profile in different tissues during *S. agalactiae* or *A. hydrophila* infection. Different letters indicate significance. Data are presented as mean ± SD (*n* = 3).

*On-CSTB* expression was significantly induced in different tissues following infection with *A. hydrophila* and *S. agalactiae. On-CSTB* expression peak was occurred at 12 h post-infection whereas peak expression was observed at 24 h in brain, spleen, and intestine following *S. agalactiae* challenge. Meanwhile, in response to *A. hydrophil* infection, *On-CSTB* expression peaked at 24 h in the brain, head kidney, spleen, and intestine, while it peaked expression at 48 h in liver (Figure 2).

### Effect of On-CSTB on SR and bacteria burden

**Figure 3.**
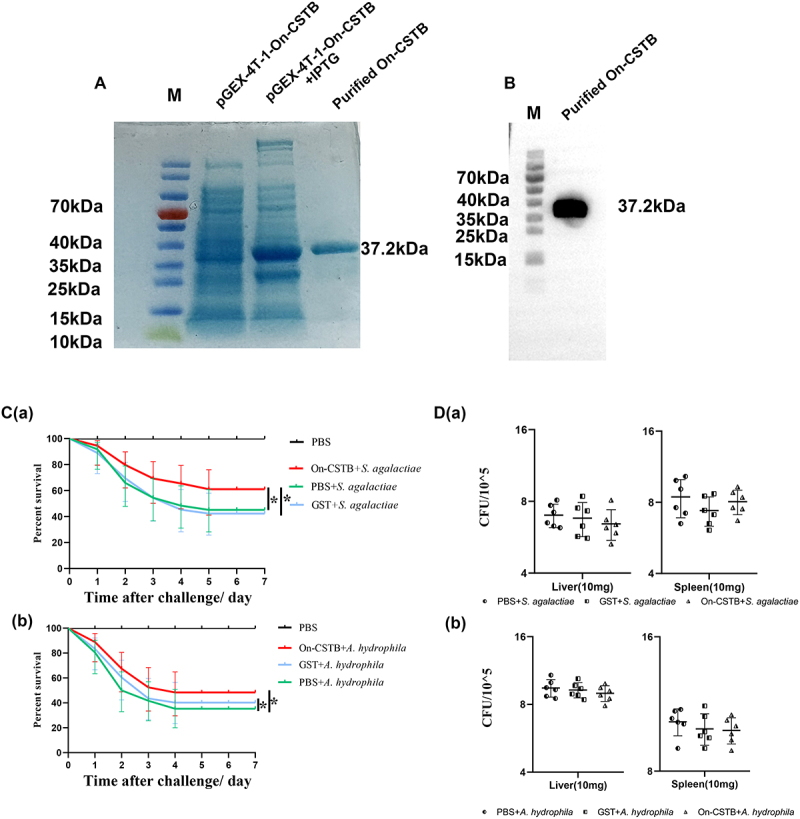
(A) SDS-PAGE analysis of recombinant protein of pGEX-4T-1-On-CSTB. Lane M, markers. (B) Western blotting of On-CSTB. (C) *S. agalactiae* (a) and *A. hydrophila* (b) infection survival rates of Nile tilapia. The daily mortality rate was recorded and analysed over a period of 7 days. Sample size: *n* = 50 per group. (D) Liver and spleen bacterial load 48 hours after *S. agalactiae* (a) and *A. hydrophila* (b) infection. Data are presented as mean ± SD (*n* = 6).

On-CSTB protein was successfully expressed with a molecular weight of 37.2 kDa (Figure 3A,B) and a tag protein size of 26 kDa (Figure S1). On-CSTB exhibited a significant effect on SR during the bacteria infection. The SR of the PBS group, *S. agalactiae* + On-CSTB group, *S. agalactiae* + GST group, *S. agalactiae* + PBS group, *A. hydrophila*+ On-CSTB group, *A. hydrophila* + GST group, and *A. hydrophila* + PBS group were 100%, 66%, 46%, 43%, 55%, 38%, and 35%, respectively (Figure 3C). However, the bacteria burden analysis revealed no significant differences in bacterial loads in the spleen and liver (Figure 3D).

### Effect of On-CSTB on histologic features during bacterial infection

**Figure 4.**
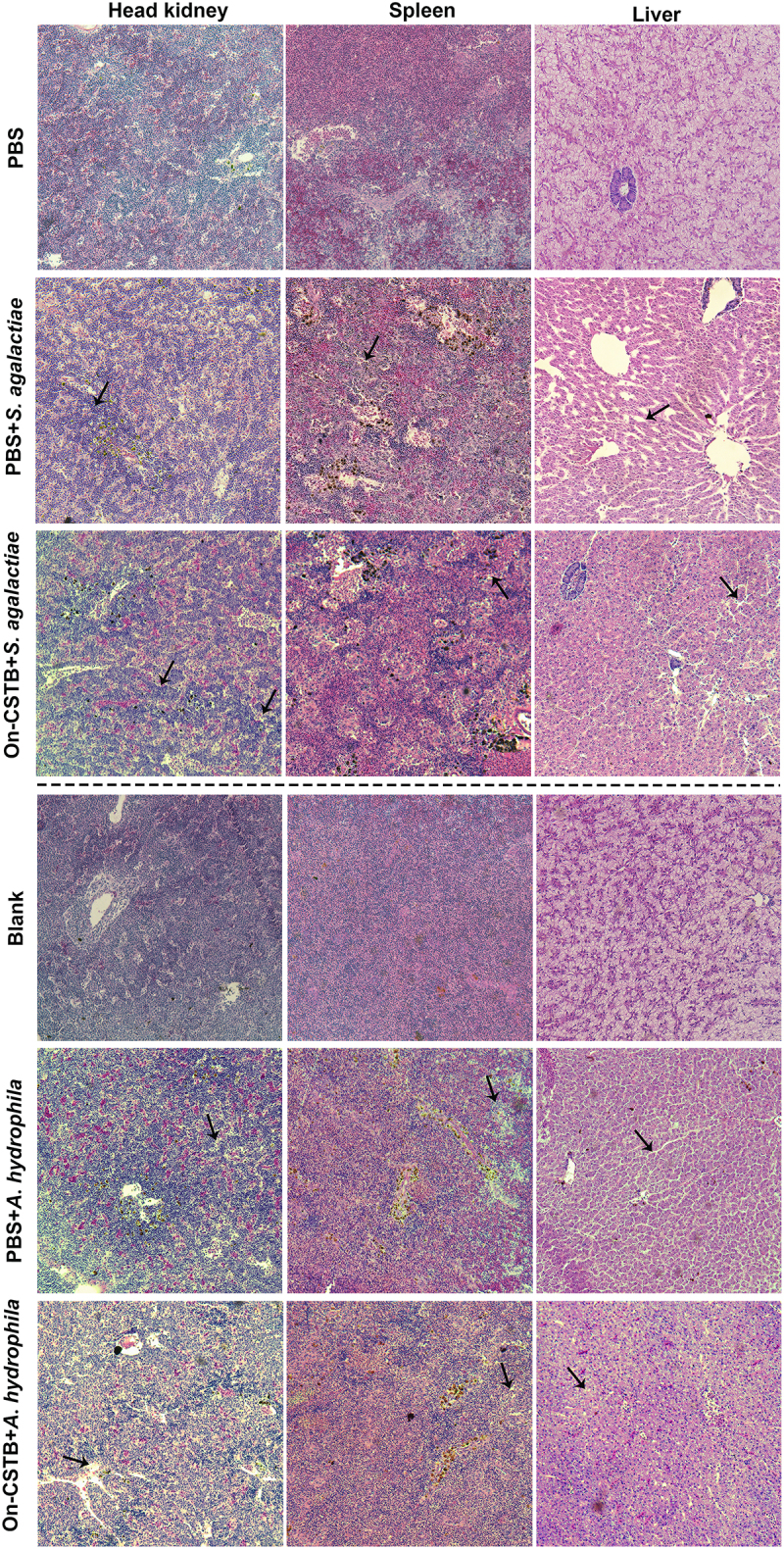
Histological analysis during *S. agalactiae* and *A. hydrophila* infection. The tissues were stained with H&E (200× magnification) and arrows indicate necrotic foci or nuclei.

The H&E staining revealed varying degrees of pathological changes across the tissue sections. In the On-CSTB group, the head kidney, spleen, and liver exhibited more intact tissue structures with closely packed cells, in contrast to the PBS+*S. agalactiae* or PBS+*A. hydrophila* group which pronounced pathological changes including tissue fragmentation and nuclear accumulation, were observed (Figure 4).

### Effect of On-CSTB on inflammatory factors during bacterial infection

**Figure 5.**
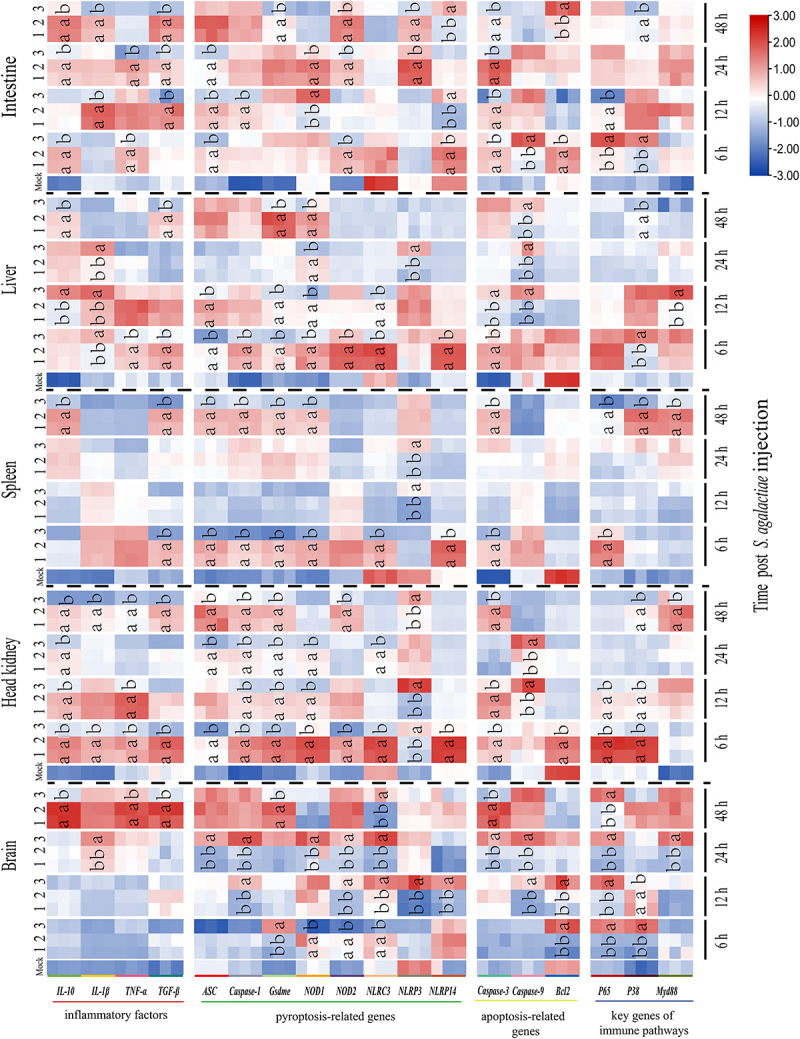
Expression profiles of genes associated with the immune system and apoptosis/pyroptosis at different time points during *S. agalactiae* infection (1: *S. agalactiae* + PBS; 2: *S. agalactiae* + GST; 3: *S. agalactiae* + On-CSTB). Data are presented as mean ± SD (*n* = 3). Statistical analysis using one-way ANOVA to determine significance and different letters indicate significance (*p* <0.05).

**Figure 6.**
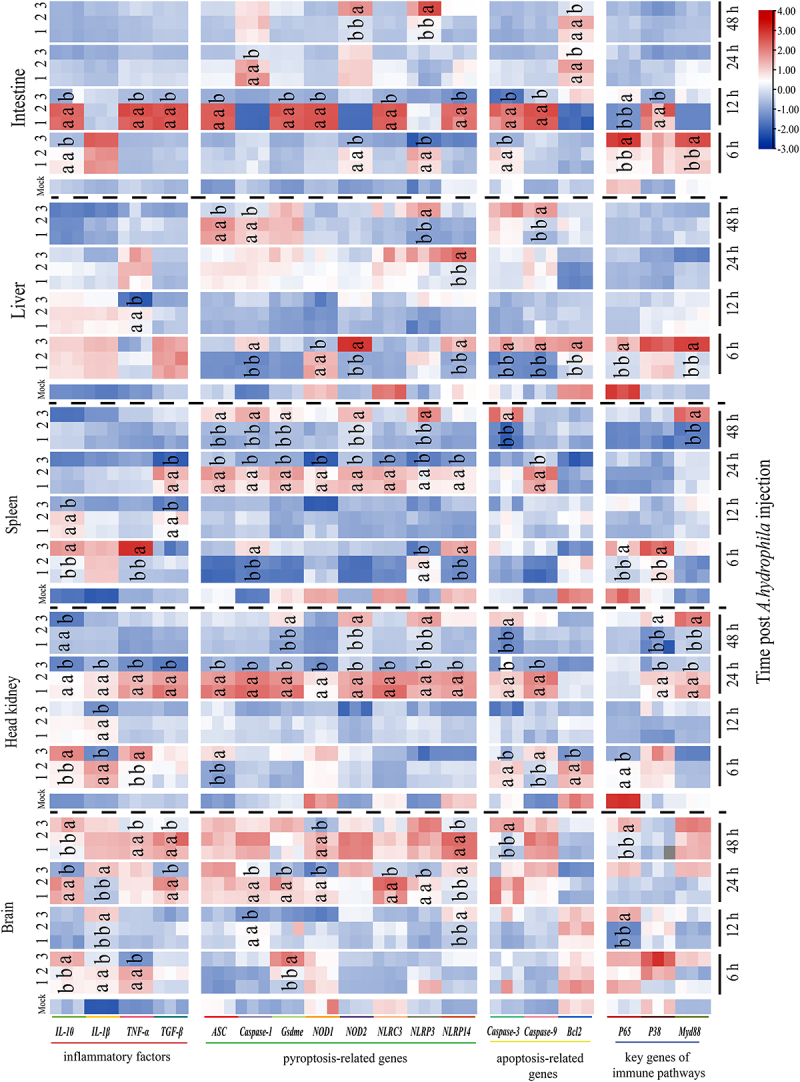
Expression profiles of genes associated with the immune system and apoptosis/pyroptosis at different time points during *A. hydrophila* infection (1: *A. hydrophila* + PBS; 2: *A. hydrophila* + GST; 3: *A. hydrophila* + On-CSTB). Data are presented as mean ± SD (*n* = 3). Statistical analysis using one-way ANOVA to determine significance and different letters indicate significance (*p* <0.05).

The function of On-CSTB on inflammatory factors expression during bacterial infection was further detected via qPCR. On-CSTB could significantly decrease (*p* < 0.05) the *interleukin-1 beta* (*IL-1β*), *tumor necrosis factor-alpha* (*TNF-α*), *interleukin-10* (*IL-10*), and *transforming growth factor-beta* (*TGF-β*) expression compared with the GST and PBS groups during *S. agalactiae* and *A. hydrophila* infection (Figures 5 and 6). Interestingly, *IL-10* expression was increased in the On-CSTB group at certain times (*p* < 0.05) compared with PBS and GST groups.

### Effect of On-CSTB on apoptosis and pyroptosis during bacterial infection

The apoptosis-related genes were remarkably regulated by On-CSTB. Compared to the GST+ *S. agalactiae* groups and PBS+ *S. agalactiae*, the *Caspase 3* was significantly decreased in the tested tissues. Except for the decrease in expression of *Caspase 9* in the spleen and *B-cell lymphoma 2* (*Bcl2*) in the head kidney, On-CSTB significantly upregulated (*p* < 0.05) *Caspase 9* and *Bcl2* expression in other tissues compared with GST+ *S. agalactiae* and PBS+ *S. agalactiae* (Figure 5). Meanwhile, compared to *A. hydrophila* + GST group and *A. hydrophila* + PBS group, the *Caspase 3* and *9* were remarkably decreased in the head kidney, spleen, and intestine and the *Bcl2* was almost decreased (*p* < 0.05) in all tissues in On-CSTB group (Figure 6).

Further, the pyroptosis-related genes including *apoptosis-associated speck-like protein containing a CARD* (*ASC*), *Caspase 1, Gasdermin-E* (*Gsdme*), *nucleotide-binding oligomerization domain-containing protein 1*(*NOD1*), *nucleotide-binding oligomerization domain-containing protein 2* (*NOD2*), *NOD-like receptor family CARD domain-containing 3* (*NLRC3*), *NOD-like receptor family pyrin domain-containing 3* (*NLRP3*), and *NOD-like receptor family pyrin domain containing 14* (*NLRP14*) were also assessed. Compared with the PBS+ *S. agalactiae* and GST+ *S. agalactiae* or *A. hydrophila* + PBS group and *A. hydrophila* + GST group, the pyroptosis-related genes were generally down-regulated (*p* < 0.05) by On-CSTB in the detected tissues. However, an opposite trend was observed in the brain following *S. agalactiae* challenge (Figures 5 and 6).

### Impact of On-CSTB on immune pathways during bacterial infection

The *nuclear factor NF-kappa-B p65 subunit* (*P65*), *mitogen-activated protein kinase 14* (*P38*), and *myeloid differentiation primary response protein 88* (*MyD88*) were significantly increased by On-CSTB in brain after *S. agalactiae* and *A. hydrophila* infection. And the contrast results were observed in head kidney, spleen, liver, and intestine which On-CSTB could remarkable decreased (*p* < 0.05) the *P65*, *P38*, and *MyD88* expression, except at a few specific time points (Figures 5 and 6).

## Discussion

Cystatin B, an endogenous protease inhibitor, plays a critical role in regulating various physiological processes, particularly in modulating the host’s innate immune defense against pathogenic infections. While the function of cystatin B has been extensively studied in mammals [12,13], its role in lower vertebrate fish has only recently gained attention. However, its specific involvement in immune responses to bacterial infections remains poorly understood. In this study, a cystatin B (CSTB) homolog from Nile tilapia was identified and characterized.

On-CSTB is a protein of 99 amino acids with a molecular weight of 11.2 kDa, which share over 75% identity with CSTB from other fish species and approximately 45% identity with mammalian CSTB, suggesting a high degree of evolutionary conservation. Meanwhile, On-CSTB exhibited the highest transcription levels in blood cells, whereas the lowest expression was detected in the head kidney. However, in orange-spotted grouper (*Epinephelus coioides*) [27], korean rockfish (*Sebastes schlegelii*) [28], and silver pomfret (*Pampus argenteus*) [10], and turbot (*Scophthalmus maximus*) [8], the highest *CSTB* transcription levels were observed in the gill or head kidney, suggesting potential evolutionary divergence of CSTB function among fish lineages, particularly between marine and freshwater species.

After infection with the *S. agalactiae* and *A. hydrophila*, On-CSTB was significantly up-regulated in different tissues, especially in the organs rich in immune cells, like head kidney, spleen, and intestine. Additionally, On-CSTB expression peaked at 12 h post-*S. agalactiae* infection, whereas its peak expression was observed later, at 24 h, following *A. hydrophila* infection. Similar result could be observed that *cystatin B* from *Pampus argenteus* was found to be significantly up-regulated in the kidney after infection with *Vibrio parahaemolyticus* [10]. In *Oplegnathus fasciatus*, *cystatin B* was also up-regulated in spleen and head kidney after *Edwardsiella tarda* challenge [15]. Moreover, *Streptococcus iniae* and lipopolysaccharides (LPS) both significantly induced *CSTB* expression in peripheral blood cells (PBCs) of big-belly seahorse (*Hippocampus abdominalis*) [29]. Overall, the present results suggested that *On-CSTB* plays an important role in innate immunity in fish, especially in resistance to pathogen invasion.

To further investigate the CSTB-mediated immune response to bacterial infection, a On-CSTB recombinant protein was prepared in in vivo study. Our results demonstrated that On-CSTB could significantly enhance the SR of Nile tilapia following gram-positive and gram-negative bacteria infection. In contrast, stefin B deficient mice showed a lower SR compared with wild-type mice after being injected with LPS [12]. However, On-CSTB exhibited a limited effect on reducing bacterial burden in infected tissues. Meanwhile, tissue section analysis revealed that On-CSTB improved tissue integrity and reduced pathological damage compared to the bacterial infection groups. Similarly, mice injected with cystatin after colitis showed reduced colon shrinkage compared to untreated colitis mice [25]. These findings suggest that On-CSTB may play a role in tissue repair by mitigating pathological damage and preserving tissue integrity.

Meanwhile, On-CSTB significantly modulated inflammatory factors expression during the *S. agalactiae* and *A. hydrophila* infection. Our results indicated that On-CSTB can significantly decrease the pro-inflammatory factors *IL-1β* and *TNF-α*, as well as the anti-inflammatory cytokine *TGF-β*. However, On-CSTB only up-regulated *IL-10* in individual time point after *S. agalactiae* infection while the On-CSTB could enhanced the *IL-10* at early time (6 h) after *A. hydrophila* infection. Similarly, CSTB from barber pole worm (*Haemonchus contortus*) could decrease the production of TNF-α and IL-1β while enhancing IL-10 secretion in goat monocytes [30]. In LPS-induced sepsis mice, *stefin B*-deficient mice exhibited increased IL-1β secretion and TNF-α release was only significantly elevated at the 2-hour time point [12]. In addition, *stefin B* deficient microglia result showed higher pro-inflammatory M1 macrophages and anti-inflammatory M2 macrophages microglia compared to control mice [13]. Another study further demonstrated that stefin B deficiency led to decreased IL-10 expression in bone marrow-derived macrophage (BMDMs) [31]. Overall, these findings indicate that CSTB is crucial in regulating inflammatory mediators.

In mammals, extensive studies have shown that inhibiting CSTB could induce apoptosis [12,32–34]. Apoptosis is a highly controlled process of programmed cell death that plays an important role in maintaining cellular homeostasis and immune regulation [35,36] while pyroptosis is a lytic form of inflammatory caspase-mediated programmed cell death triggered by various pathological stimuli [37,38]. However, a bidirectional interplay between apoptosis and pyroptosis exists, wherein activated caspase-1 can cleave and activate caspase-3 and −7, thereby further amplifying the inflammatory response [39]. This crosstalk not only strengthens cell death signaling but may also be conducive to the production of proinflammatory cytokines, like *IL-1β* and *IL-18*, that may additionally aggravate the immune response. In fish, it has been identified that NLRP3-dependent pyroptosis pathways as the predominant pyroptotic mechanism [40]. Moreover, our previous study also declared NLRCs and pyroptosis-related genes involved in the immune response in Nile tilapia [41]. Thus, in this study, three NLRCs, five pyroptosis-related genes, and three apoptosis genes were subsequently were assessed and analyzed. Our results demonstrated that On-CSTB significantly downregulated NLRCs and pyroptosis-related genes, except for an increase observed in the brain following *S. agalactiae* and *A. hydrophila* infection. Additionally, apoptosis-related genes were generally suppressed, while *caspase-9* was upregulated at specific time points. Similar result was found that *cystatin B*-deficient mice result in cerebellar granule cell apoptosis [42]. Additionally, *cystatin B*-deficient thymocytes exhibit increased sensitivity to apoptosis induced by the staurosporin [43]. Typically, apoptosis is often regarded as a more delayed and tightly regulated form of cell death compared to the rapid and inflammatory nature of pyroptosis [44–46]. Thus, these findings suggest that *On-CSTB* involved in the innate immune response of fish, as well as the regulation of inflammasome activation and pyroptosis.

The Toll-like receptor (TLR)-mediated MyD88/nuclear factor kappa B (NF-κB) signaling pathway is a canonical inflammatory signaling pathway in organisms, playing a crucial role in immune defense and inflammation regulation during pathogen infection or tissue damage [47,48]. It has been reported that *CSTB* overexpression in macrophage reporter cells significantly suppressed NF-κB activation under LPS stimulation [12]. Further, our data demonstrated that On-CSTB significantly down-regulated *P65*, *P38*, and *MyD88* expression in the head kidney and spleen, which are key components of the MyD88/NF-κB signaling pathway during *S. agalactiae* infection. However, On-CSTB only mainly down-regulated *P65*, *P38*, and *MyD88* at 12 and 24 h in head kidney, spleen, liver, and intestine wherever up-regulated in brain during *A. hydrophila* infection. These results suggest that On-CSTB plays a regulatory role in modulating the MyD88/NF-κB signaling pathway during bacterial infections and On-CSTB may have distinct roles in immune regulation depending on the pathogen and the affected organ.

In conclusion, we identified and characterized *cystatin B* (*On-CSTB*) from Nile tilapia and explored its role in the immune response to bacterial infection. Our study suggests that *On-CSTB* plays a crucial anti-inflammatory role by modulating immune signaling pathways, reducing excessive cell apoptosis and pyroptosis, and contributing to tissue protection. Its regulatory effects on the MyD88/NF-κB signaling pathway, as well as its involvement in inflammasome and pyroptosis regulation, highlight its potential function in maintaining immune homeostasis during bacterial infections. These results not only enhance our understanding of fish immune regulation but also provide a basis for future application in disease control strategies. However, limitations exist in this study, including the lack of in vivo functional verification using gene knockout or knockdown models. Future studies should explore the upstream regulatory network of *CSTB*, its interaction with other immune signaling molecules, and its roles in different types of immune challenges such as viral or parasitic infections. Understanding the dynamic regulation of *CSTB* under stress or environmental changes will also help in developing more precise aquaculture immunomodulatory strategies.

## Supplementary Material

Figure S1.docx

QVIR-2025-0262.R1-ARRIVE checklist.pdf

## Data Availability

The datasets presented in this study can be found in figshare:https://figshare.com/articles/dataset/Dataset/28815197?file=53743007. DOI:10.6084/m9.figshare.28815197.

## Statement on ARRIVE guidelines

This study was designed, conducted, and reported in accordance with the ARRIVE (Animal Research: Reporting of In Vivo Experiments) guidelines to ensure transparency, reproducibility, and ethical standards in animal research.
