# Supplementary material for: Pathological protection and molecular regulation of cystatin B in Nile tilapia (Oreochromis niloticus) bacterial disease
Source: Virulence. 2025 Sep 29;16(1):2563010. doi: 10.1080/21505594.2025.2563010 (PMC12482433; doi:10.1080/21505594.2025.2563010)
Supplement: Figure S1.docx [file KVIR_A_2563010_SM2875.docx]

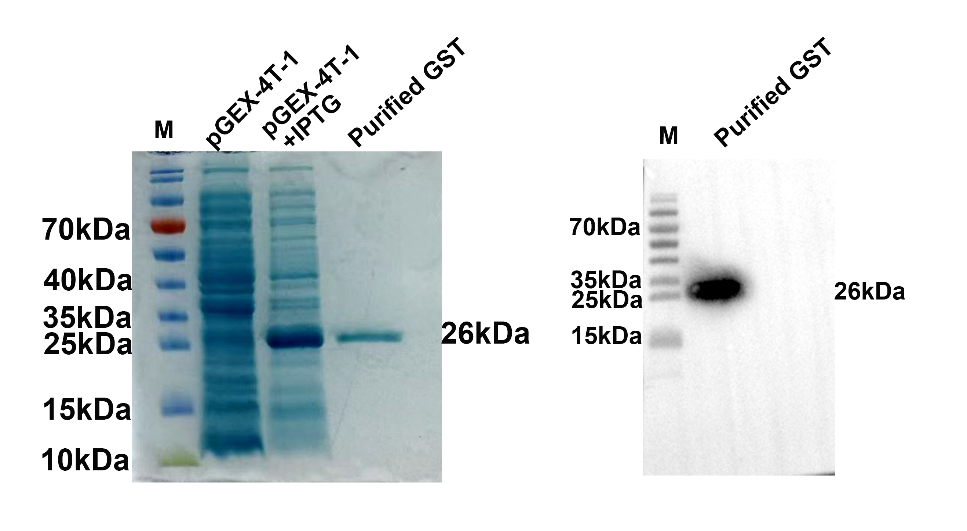


Figure S1: SDS-PAGE was performed on the total protein content of both the uninduced and induced strains, as well as on the purified protein carrying the GST-tag.
